# Using machine learning for mortality prediction and risk stratification in atezolizumab‐treated cancer patients: Integrative analysis of eight clinical trials

**DOI:** 10.1002/cam4.5060

**Published:** 2022-07-24

**Authors:** Yougen Wu, Wenyu Zhu, Jing Wang, Lvwen Liu, Wei Zhang, Yang Wang, Jindong Shi, Ju Xia, Yuting Gu, Qingqing Qian, Yang Hong

**Affiliations:** ^1^ National Institute of Clinical Research, The Fifth People's Hospital of Shanghai Fudan University Shanghai China; ^2^ Shanghai Long For Health Data Technology Co.ltd Shanghai China; ^3^ Department of Biostatistics Fudan University School of Public Health Shanghai China; ^4^ Department of Urology The Fifth People's Hospital of Shanghai, Fudan University Shanghai China; ^5^ Department of Respiratory Medicine The Fifth People's Hospital of Shanghai, Fudan University Shanghai China; ^6^ Department of Pharmacy, The Fifth People's Hospital of Shanghai Fudan University Shanghai China; ^7^ Department of Osteology, The Fifth People's Hospital of Shanghai Fudan University Shanghai China

**Keywords:** cancer immunotherapy, machine learning, mortality prediction, risk stratification

## Abstract

**Background:**

Few models exist to predict mortality in cancer patients receiving immunotherapy. Our aim was to build a machine learning‐based risk stratification model for predicting mortality in atezolizumab‐treated cancer patients.

**Methods:**

Data from 2538 patients in eight atezolizumab‐treated cancer clinical trials across three cancer types (non‐small‐cell lung cancer, bladder transitional cell carcinoma, and renal cell carcinoma) were included. The whole cohort was randomly split into development and validation cohorts in a 7:3 ratio. Machine‐learning algorithms (extreme gradient boosting, random forest, logistic regression with lasso regularization, support vector machine, and K‐nearest neighbor) were applied to develop prediction models. Model performance was mainly assessed by area under the receiver operating characteristic curve (AUC) value, calibration plot, and decision curve analysis. The probability of death risk was then stratified.

**Results:**

One thousand and three hundred and seventy‐nine (54.33%) patients died. The random forest (RF) model was overall the best in terms of predictive performance, with the AUC of 0.844 (95% confidence interval [CI]: 0.826–0.862) in the development cohort and 0.786 (95% CI: 0.754–0.818) in the validation cohort for predicting mortality. Twelve baseline variables contributing to mortality prediction in the RF model were C‐reactive protein, PD‐L1 level, cancer type, prior liver metastasis, derived neutrophil‐to‐lymphocyte ratio, alkaline phosphatase, albumin, hemoglobin, white blood cell count, number of metastatic sites, pulse rate, and Eastern Cooperative Oncology Group (ECOG) performance status. A total of 1782 (70.2%) patients were separated into the high‐risk and 756 (29.8%) low‐risk groups. Patients in the high‐risk group were significantly more likely to die, experience disease progression, discontinue study, and discontinue treatment than patients in the low‐risk group (all *p* values < 0.001). Risk groups were not associated with immune‐related adverse events and grades 3–5 treatment‐related adverse events (all *p* values > 0.05).

**Conclusion:**

RF model has good performance in mortality prediction and risk stratification for cancer patients receiving atezolizumab monotherapy.

## Graphical abstract

Random forest model with 12 variables could be a useful tool to identify individuals who are most likely to have adverse outcomes (death, disease progression, discontinued study, or discontinued treatment) and clinical benefit from atezolizumab monotherapy. Laboratory tests, and tumor characteristics evaluation performed at baseline before starting immunotherapy may provide an important reference for weighing benefits and risks of therapy.

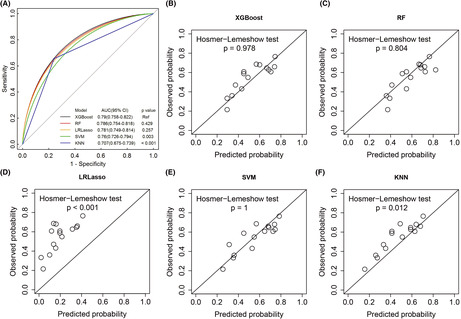

## 1 INTRODUCTION

Immunotherapy with immune checkpoint inhibitors (ICIs) has been used in the treatment of various cancer types, including advanced melanoma, non‐small‐cell lung carcinoma (NSCLC), urothelial cancer, and hepatocellular carcinoma.^1^ Atezolizumab selectively targets programmed death ligand 1 (PD‐L1) and prevents the interactions of PD‐L1 with programmed death‐1 (PD‐1) and B7‐1, which can reinvigorate and stimulate anticancer immunity. Atezolizumab is approved for metastatic non‐small‐cell lung cancer, small‐cell lung cancer, melanoma, and advanced triple‐negative breast cancer and in combination with bevacizumab for unresectable hepatocellular carcinoma.^2^, ^3^

Despite durable response and improved survival outcomes, ICIs benefit a minority of patients. Only 10%–40% of patients with advanced solid tumors respond to ICIs treatment.^4^ Additionally, activation of the immune system by ICIs can lead to inflammatory side effects, which are often known as immune‐related adverse events (irAEs).^5^ IrAEs can result in either temporary treatment interruption or dose reduction and/or discontinuation of ICIs therapy.^6^

Although various clinical factors related to immunotherapy benefits and risk have been identified, such as PD‐L1 expression, tumor mutational burden (TMB), microsatellite instability‐high (MSI‐H), and neutrophil‐to‐lymphocyte ratio, none of these factors can alone accurately identify individuals who are most likely to have adverse outcomes and clinical benefit from immunotherapy.^7^, ^8^, ^9^, ^10^, ^11^

Machine learning (ML) is increasingly explored to predict clinical events, such as acute kidney injury, cardiovascular risk, and fracture risk, with good results.^12^, ^13^, ^14^ ML approaches may enable to overcome some of the limitations of conventional statistical methods in risk prediction via using algorithms to evaluate large data sets with numerous features, capturing complex multidimensional correlations between features and clinical outcomes.^15^

Our primary objective was to build an ML‐based risk stratification model based on clinical trial data for mortality prediction in cancer patients treated with atezolizumab monotherapy.

## 2 PATIENTS AND METHODS

### 2.1 Source of data and study population

Deidentified, patient‐level data of eight randomized clinical trials (NCT01846416, NCT01903993, NCT02031458, NCT02008227, NCT02951767, NCT02108652, NCT02302807, and NCT01984242) across three cancer types (non‐small‐cell lung cancer, bladder transitional cell carcinoma, and renal cell carcinoma) from Roche were assessed from the Vivli platform (www.vivli.org). Details of eight clinical trials used for this study are shown in Table S1. Patients included in this study were those who received atezolizumab monotherapy during the study treatment period. Patients who did not receive atezolizumab treatment or who received atezolizumab in combination with bevacizumab treatment were excluded.

### 2.2 Predictor features and outcomes

All the predictors are baseline characteristics data from clinical trials. Features were derived from six key domains: patient demographics, medical history, concomitant medication, laboratory test results, ECG test results, and vital signs results. We dropped the variables with more than 35% missing data. The original variables may be transformed, including but not limited to converting continuous variables to categorical variables, and combining multiple variables into a single variable for information integration. The resulting data set had 235 features, among which 52 features were continuous variables and 183 were categorical variables.

The primary predicted outcome was death (i.e., “DISCDEAT,” as recorded in the data set). Secondary outcomes included progression‐free survival (PFS), patient discontinued study (i.e., “DISCSTUD”), patient discontinued treatment (i.e., “DISTRTFL”), immune‐related adverse events (i.e., “AEIMFL” and “CMIMFL”), and grade 3–5 treatment‐related adverse events (i.e., “grade 3–5 TRAES”). Grade 3–5 TRAES was derived from two factors^1^: causality for adverse events (i.e., “AEREL”) and^2^ standard toxicity grade (i.e., “AETOXGR”). According to the National Cancer Institute Common Terminology Criteria for Adverse Events (NCI CTCAE v4.0), the severity of adverse events includes grades 1 (mild), 2 (moderate), 3 (severe), 4 (life‐threatening), and 5 (death).

### 2.3 Variables selected by least absolute shrinkage and selection operator (LASSO)

The LASSO method, which is suitable for the regression of high‐dimensional data, was applied to select the most predictive variables by eliminating redundant collinear variables.^16^ The lambda with one standard error (SE) of the minimum cross‐validated error was used for variable selection.

### 2.4 Data preprocessing

Missing data for each variable were imputed via the Multivariate Imputation by Chained Equations (MICE) methods. *Z*‐score standardization was conducted after imputation as the range of different numeric variables varied widely.^17^

### 2.5 Machine‐learning model development and validation

Model development, validation, and reporting adhered to the Transparent Reporting of a multivariable prediction model for Individual Prognosis or Diagnosis (TRIPOD) guideline.^18^ The cohort was split randomly into the development (70%) cohort and validation (30%) cohort according to the classical split‐sample internal validation approach.^18^ The development cohort was utilized to train ML models and tune their parameters, whereas the validation cohort was used to evaluate the performance of developed models on unseen data.

We developed five ML models—extreme gradient boosting (XGBoost), random forest (RF), logistic regression with lasso regularization (LRLasso), support vector machine (SVM), and K‐nearest neighbor (KNN)—to predict the risk of mortality. The best‐performing model was internally fivefold cross‐validated to assess whether or not the model is overfitting.

### 2.6 Model evaluation

Model performance was evaluated by receiver operating characteristic (ROC) curve, calibration plot, Hosmer‐Lemeshow goodness‐of‐fit test, and additional evaluation metrics comprising area under the ROC curve (AUC), accuracy, sensitivity, specificity, positive predictive value (PPV), negative predictive value (NPV), F1 score, Cohen's Kappa coefficient (Kappa), and Brier score.^19^ Differences in the AUC values between various models were compared by using the DeLong test. Decision curve analysis (DCA) was performed to calculate the net benefit of each prediction model.^20^

### 2.7 Feature importance and partial dependence plots

The importance of different features on the outcome in the model was calculated by varImpPlot function in the random forest R package.

Partial dependence plots (PDPs) provide insight into black‐box ML models, visualizing how each variable affects prediction while averaging over all the other features.^21^, ^22^ PDPs show the marginal effect of each feature on response probability.^22^

### 2.8 Risk stratification using ML

The death prediction task was treated as a binary classification problem. ML models output a probability of death risk ranging from 0 to 1. We used risk probabilities calculated by the best‐performing ML model to define the optimal cutoff values that best stratify patients into two risk groups (low and high) by maximizing the F1 score. Survival probabilities of the risk groups were evaluated by the Kaplan–Meier method.

### 2.9 Statistical analysis

Statistical analysis was conducted in R (version 3.5.2). Categorical variables were reported as frequencies (%) and continuous variables as median (interquartile range). We used the chi‐square test to calculate differences at baseline between groups for categorical variables, and the Mann–Whitney U test for non‐parametric variables. A two‐tailed p value below 0.05 was regarded as statistically significant.

In this research, R packages “haven,” “reshape2,” “tidyverse,” “mice,” “randomForest,” “xgboost,” “glmnet,” “e1071,” “class,” “caret,” “plotmo,” “ResourceSelection,” “dca.R,” “finalfit,” “ggplot2,” “Rmisc,” “rms,” “pROC,” “survminer,” and “survival” were used for data preprocessing and statistical analysis.

## 3 RESULTS

### 3.1 Baseline characteristics of the patients

Baseline characteristics of the development and validation cohorts are summarized in Table 1. According to the inclusion and exclusion criteria, a total of 2538 patients across three different cancer types (non‐small‐cell lung cancer, bladder transitional cell carcinoma, and renal cell carcinoma) were included in the present study. Among these patients, 1379 (54.3%) died and 1159 (45.7%) did not. Of the 2538 eligible patients, 1547 (61.0%) were non‐small‐cell lung cancer, 888 (35.0%) were bladder transitional cell carcinoma, and 103 (4.1%) were renal cell carcinoma. The majority of patients were White (79.7%), male (67.0%), and had smoking history (74.2%). In addition, 1522 (60.0%) patients presented with Eastern Cooperative Oncology Group (ECOG) performance status ≥1, 1402 (55.2%) had less than three metastatic sites, 1308 (51.5%) had high PD‐L1 expression, and 1196 (47.1%) patients had stage IV cancer. The development cohort included more patients with abnormal alkaline phosphatase compared with the validation cohort (*p* = 0.028). Distributions of other baseline characteristics were similar in the two groups of patients (all *p* values > 0.05, Table 1).

**TABLE 1:** Comparison of baseline characteristics between patients in the development and validation cohorts

Variable	Levels	Development cohort (*n* = 1776)	Validation cohort (*n* = 762)	Total (*n* = 2538)	*p* value
Age (years)	Median (IQR)	65.0 (58.0 to 71.0)	66.0 (57.0 to 72.0)	65.0 (58.0 to 72.0)	0.311
Body mass index (kg/m^2^)	Median (IQR)	25.5 (22.5 to 28.9)	25.5 (22.7 to 28.7)	25.5 (22.5 to 28.8)	0.653
Sex	Female	588 (33.1)	249 (32.7)	837 (33.0)	0.868
	Male	1188 (66.9)	513 (67.3)	1701 (67.0)	
Race	White	1401 (78.9)	622 (81.6)	2023 (79.7)	0.166
	Asian	221 (12.4)	83 (10.9)	304 (12.0)	
	Black or African American	38 (2.1)	8 (1.0)	46 (1.8)	
	Other or Unknown	116 (6.5)	49 (6.4)	165 (6.5)	
Smoking history	Never	388 (21.8)	163 (21.4)	551 (21.7)	0.725
	Current or Previous	1309 (73.7)	574 (75.3)	1883 (74.2)	
	(Missing)	79 (4.4)	25 (3.3)	104 (4.1)	
Cancer type	BTCC	614 (34.6)	274 (36.0)	888 (35.0)	0.383
	NSCLC	1084 (61.0)	463 (60.8)	1547 (61.0)	
	renal	78 (4.4)	25 (3.3)	103 (4.1)	
ECOG PS	0	623 (35.1)	290 (38.1)	913 (36.0)	0.23
	≥1	1075 (60.5)	447 (58.7)	1522 (60.0)	
	(Missing)	78 (4.4)	25 (3.3)	103 (4.1)	
Tumor stage	Other	774 (43.6)	315 (41.3)	1089 (42.9)	0.338
	IV	827 (46.6)	369 (48.4)	1196 (47.1)	
	(Missing)	175 (9.9)	78 (10.2)	253 (10.0)	
No. of metastatic sites	<3	975 (54.9)	427 (56.0)	1402 (55.2)	0.804
	>2	701 (39.5)	299 (39.2)	1000 (39.4)	
	(Missing)	100 (5.6)	36 (4.7)	136 (5.4)	
Prior liver metastasis	No	1372 (77.3)	588 (77.2)	1960 (77.2)	1
	Yes	404 (22.7)	174 (22.8)	578 (22.8)	
PD‐L1 level	Low^a^	841 (47.4)	378 (49.6)	1219 (48.0)	0.345
	High	926 (52.1)	382 (50.1)	1308 (51.5)	
	(Missing)	9 (0.5)	2 (0.3)	11 (0.4)	
Derived neutrophil/lymphocyte ratio	<3	1406 (79.2)	615 (80.7)	2021 (79.6)	0.453
	≥3	338 (19.0)	135 (17.7)	473 (18.6)	
	(Missing)	32 (1.8)	12 (1.6)	44 (1.7)	
Alkaline phosphatase	Low^b^	18 (1.0)	2 (0.3)	20 (0.8)	0.028
	Normal	1320 (74.3)	594 (78.0)	1914 (75.4)	
	High^c^	398 (22.4)	147 (19.3)	545 (21.5)	
	(Missing)	40 (2.3)	19 (2.5)	59 (2.3)	
Albumin (g/L)	≤36	535 (30.1)	217 (28.5)	752 (29.6)	0.407
	>36	1199 (67.5)	529 (69.4)	1728 (68.1)	
	(Missing)	42 (2.4)	16 (2.1)	58 (2.3)	
Hemoglobin	Low^b^	1022 (57.5)	431 (56.6)	1453 (57.2)	0.796
	Normal	711 (40.0)	313 (41.1)	1024 (40.3)	
	High^c^	11 (0.6)	6 (0.8)	17 (0.7)	
	(Missing)	32 (1.8)	12 (1.6)	44 (1.7)	
White blood cell count	Low^b^	60 (3.4)	21 (2.8)	81 (3.2)	0.706
	Normal	1390 (78.3)	603 (79.1)	1993 (78.5)	
	High^c^	294 (16.6)	126 (16.5)	420 (16.5)	
	(Missing)	32 (1.8)	12 (1.6)	44 (1.7)	
C‐reactive protein (mg/L)	Median (IQR)	15.0 (4.7 to 42.3)	13.7 (4.3 to 40.0)	14.5 (4.6 to 41.7)	0.301
Pulse rate (beats/min)	Median (IQR)	82.0 (72.0 to 92.0)	82.0 (72.0 to 93.0)	82.0 (72.0 to 93.0)	0.492
ECG results	Normal	1013 (57.0)	438 (57.5)	1451 (57.2)	0.923
	Abnormal	733 (41.3)	313 (41.1)	1046 (41.2)	
	(Missing)	30 (1.7)	11 (1.4)	41 (1.6)	
Number of comorbidities	≤1	948 (53.4)	430 (56.4)	1378 (54.3)	0.17
	>1	828 (46.6)	332 (43.6)	1160 (45.7)	
Statins use	No	1676 (94.4)	722 (94.8)	2398 (94.5)	0.771
	Yes	100 (5.6)	40 (5.2)	140 (5.5)	
STEROIDS Use	No	1661 (93.5)	719 (94.4)	2380 (93.8)	0.48
	Yes	115 (6.5)	43 (5.6)	158 (6.2)	
Steroids use	No	1648 (92.8)	700 (91.9)	2348 (92.5)	0.463
	Yes	128 (7.2)	62 (8.1)	190 (7.5)	
Vital status	Alive	815 (45.9)	344 (45.1)	1159 (45.7)	0.763
	Dead	961 (54.1)	418 (54.9)	1379 (54.3)	

Abbreviations: BTCC, bladder transitional cell carcinoma; ECG, electrocardiography; ECOG PS, Eastern Cooperative Oncology Group performance status; IQR, interquartile range; NSCLC, non‐small‐cell lung cancer; PD‐L1, programmed cell death ligand 1; RCC, renal cell carcinoma.

^a^ Low PD‐L1 expression indicates PD‐L1 expression on lower than 5% of tumor cells or tumor‐infiltrating immune cells.

^b^ Low indicates the value less than the reference range lower limit of each institution.

^c^ High indicates the value greater than the reference range upper limit of each institution.

### 3.2 Feature selection

In total, 235 features were extracted from the data sets of clinical trials. Among them, 12 features with nonzero coefficients were selected by the LASSO logistic regression algorithm (Figure S1A,B) and used for model constructions. These selected 12 features were C‐reactive protein (CRP), PD‐L1 level, cancer type, prior liver metastasis, derived neutrophil‐to‐lymphocyte ratio (dNLR), alkaline phosphatase (ALP), albumin (ALB), hemoglobin (HGB), white blood cell count (WBC), number of metastatic sites, pulse rate, and Eastern Cooperative Oncology Group (ECOG) performance status.

### 3.3 Performance evaluation of machine‐learning models in predicting mortality

**FIGURE 1:**
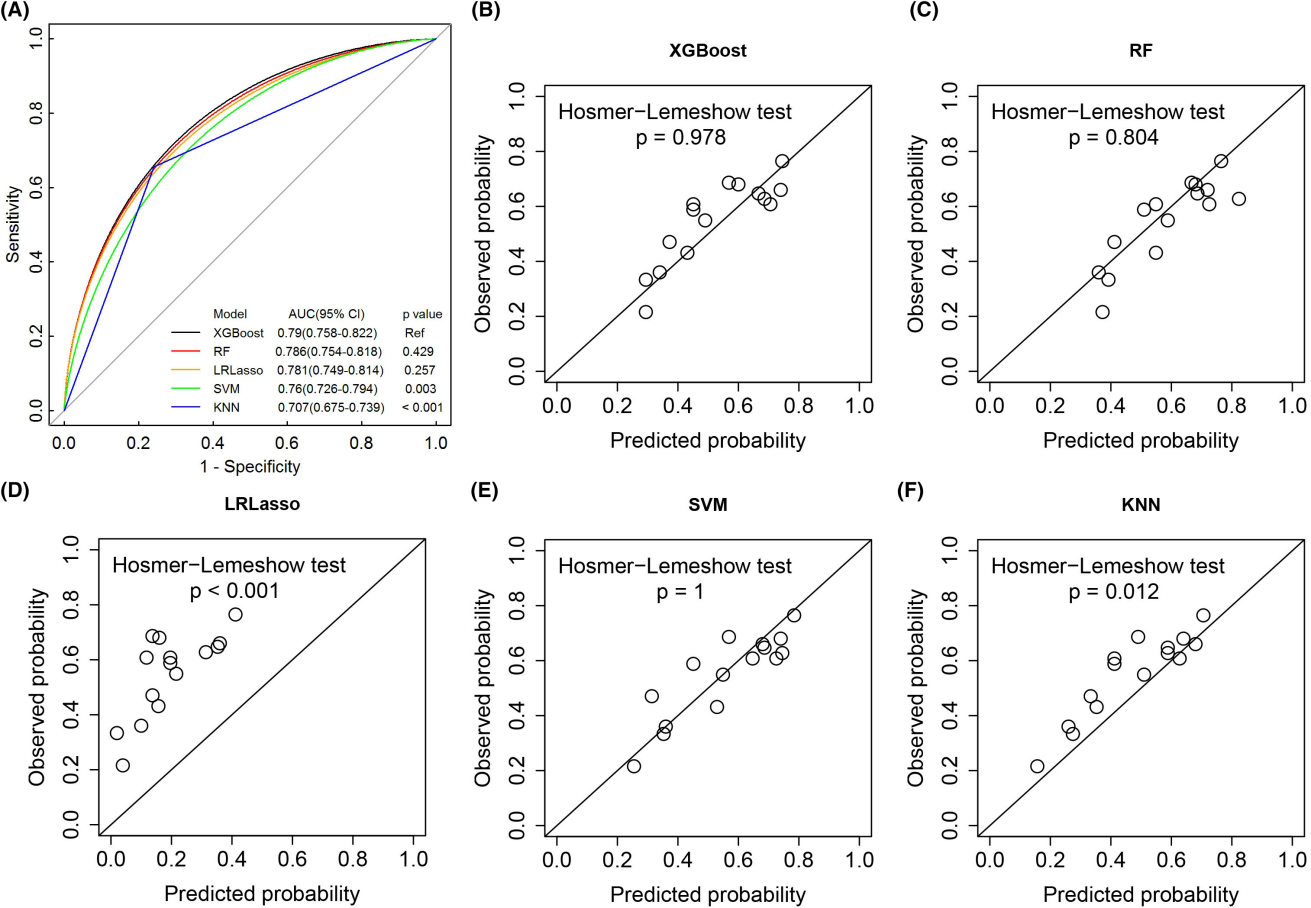
(A) ROC curves of machine‐learning models for predicting mortality on validation cohort. The higher area under the ROC curve (AUC) shows better discrimination. *p* values show the AUC for the RF model vs the AUC for other models. (B–F) Calibration plots of different machine‐learning algorithms for predicting mortality on validation cohort. The *x*‐axis is the probability of death predicted by the algorithm, and the *y*‐axis is the actual probability. The 45° straight line indicates perfect predictive ability. *p* values more than 0.05 indicate good calibration. KNN, K‐nearest neighbor; LRLasso, logistic regression with lasso regularization; Ref, reference; RF, random forest; ROC, receiver operating characteristic; SVM, support vector machine; XGBoost, extreme gradient boost.

Among the five developed models, XGBoost method yielded the highest area under the ROC curve (AUC) of 0.79 (95% confidence interval [CI]: 0.758–0.822) for predicting mortality, followed by RF model with AUC of 0.786 (95% CI: 0.754–0.818), LRLasso model with AUC of 0.781 (95% CI: 0.749–0.814), and SVM model with AUC of 0.76 (95% CI: 0.726–0.794). KNN model proved the lowest AUC of 0.707 (95% CI: 0.675–0.739) (Figure 1A).

Calibration of all the models was evaluated using the percentile plot of observed versus predicted probability and the Hosmer‐Lemeshow goodness‐of‐fit test. The XGBoost, RF, and SVM models were well calibrated (all *p* > 0.05), but both LRLasso and KNN models were not (all *p* < 0.05) (Figure 1B–F).

The predictive performances of five contributing algorithms with several metrics and confusion matrices were presented in Table 2. Using a probability cutoff of 0.5 or more to assign class, we observed that the RF model consistently obtained better predictive performance as measured by accuracy, sensitivity, negative predictive value (NPV), Kappa, F1, and Brier score compared with other models (Table 2).

**TABLE 2:** Performance metrics of machine‐learning models in predicting mortality with a threshold of 0.5 on the validation cohort

Model	Accuracy (95% CI)	Sensitivity	Specificity	PPV	NPV	Kappa	F1	Brier
XGBoost	0.719 (0.686–0.751)	0.720	0.718	0.756	0.679	0.436	0.738	0.275
RF	0.723 (0.690–0.755)	0.782	0.651	0.732	0.711	0.437	0.756	0.271
LRLasso	0.596 (0.560–0.631)	0.309	0.945	0.872	0.529	0.237	0.456	0.396
SVM	0.690 (0.656–0.723)	0.727	0.645	0.714	0.661	0.373	0.720	0.304
KNN	0.702 (0.668–0.734)	0.656	0.759	0.767	0.644	0.408	0.707	0.292

Abbreviations: 95% CI, 95% confidence interval; KNN, K‐nearest neighbor; LRLasso, logistic regression with lasso regularization; NPV, negative predictive value; PPV, positive predictive value; RF, random forest; SVM, support vector machine; XGBoost, extreme gradient boost.

**FIGURE 2:**
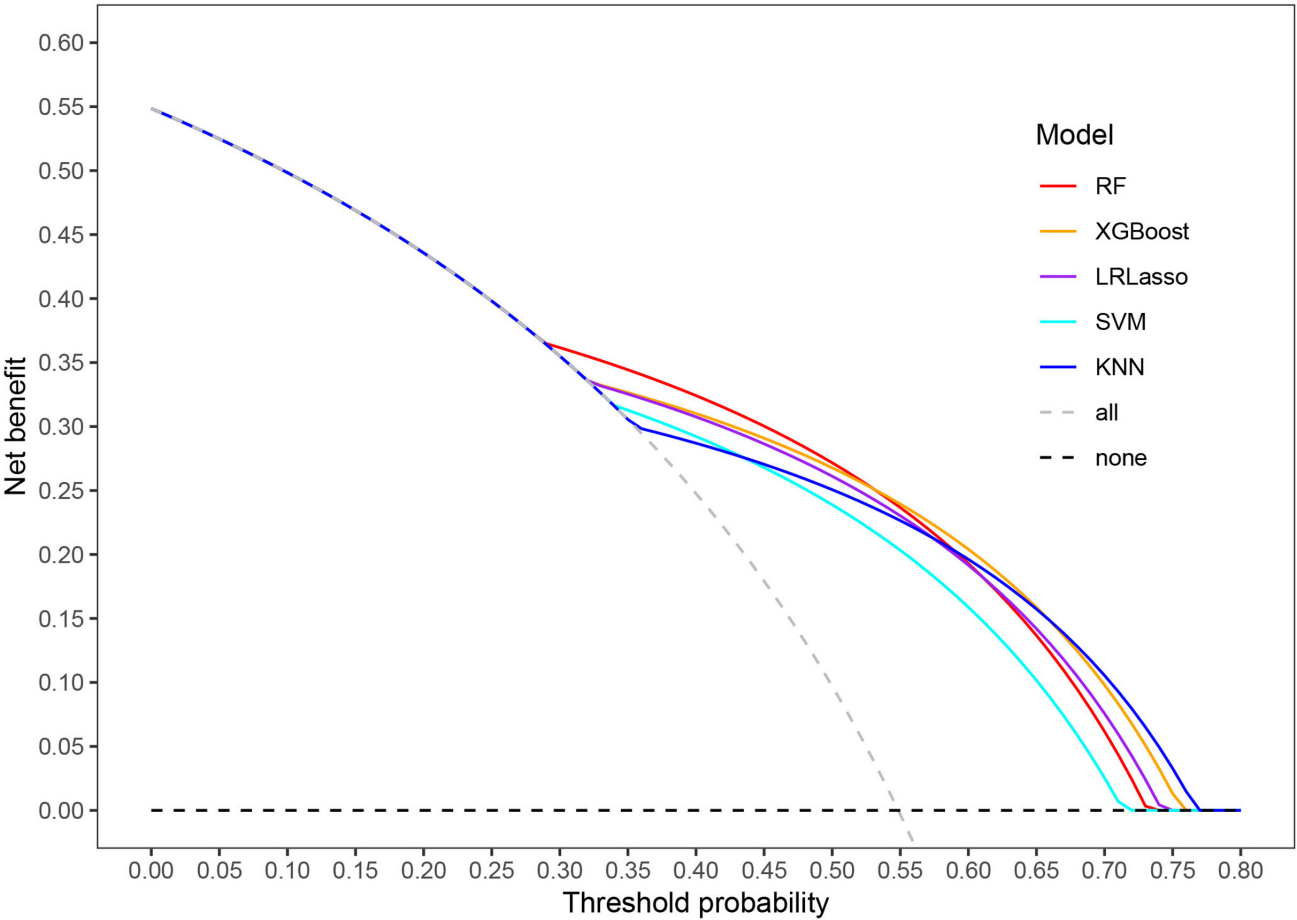
Decision curve analysis for each model in the validation cohort. The *y*‐axis measures the net benefit. The gray dashed line assumes that all patients died. The black dashed line assumes that no patient died. KNN, K‐nearest neighbor; LRLasso, logistic regression with lasso regularization; RF, random forest; SVM, support vector machine; XGBoost, extreme gradient boost.

The decision curve analysis (DCA) for each model was presented in Figure 2. All five ML‐based models showed positive net benefits in the DCA curves, with overlap among them. The gain from the RF model was particularly higher than the other four models at threshold probabilities of risk between 0.3 and 0.5. SVM model provided a lower net benefit to predict mortality than the other four models at threshold probabilities of more than 0.44 (Figure 2).

### 3.4 Ranking of variable importance for mortality prediction

**FIGURE 3:**
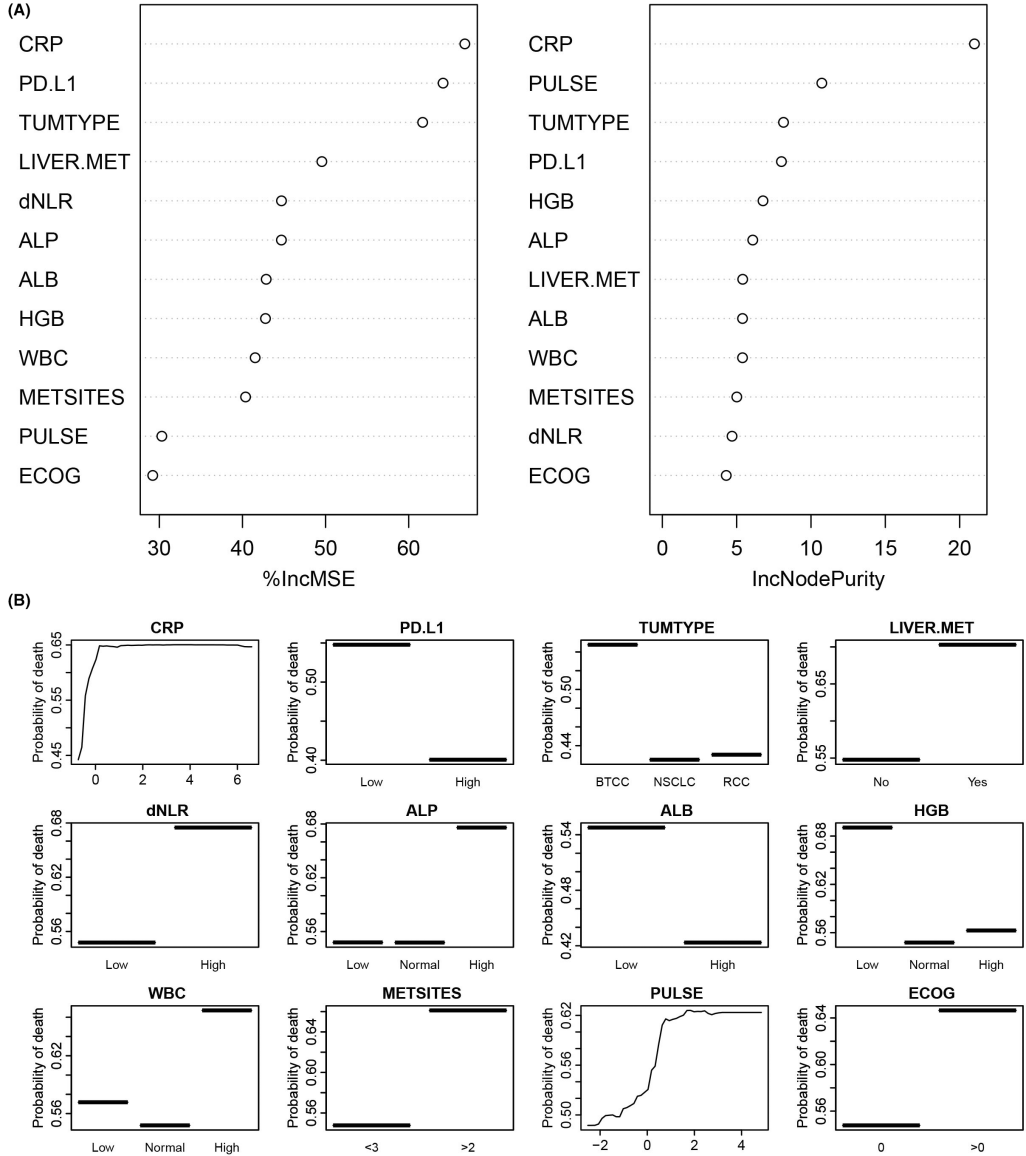
(A) The importance ranking of 12 variables calculated in the development cohort to predict mortality by random forest model. (B) Partial dependence plots of death probability and the predictors. The horizontal axis (*x*‐axis) shows values for model predictors. The vertical axis (*y*‐axis) represents the probability of death. ALB, Albumin; ALP, Alkaline phosphatase; CRP, C‐reactive protein; dNLR, Derived neutrophil‐to‐lymphocyte ratio; ECOG, Eastern Cooperative Oncology Group; HGB, Hemoglobin; IncMSE, Increase in mean square errors; IncNodePurity, Increase in Node Purity; LIVER.MET, Prior liver metastasis; METSITES, number of metastatic sites; PD.L1, PD‐L1 level; PULSE, Pulse rate; TUMTYPE, Tumor type; WBC, White blood cell count.

To assess the relative importance of each feature for mortality prediction in the model, a feature ranking analysis was conducted. CRP and tumor type were consistently ranked first and third in variable importance using two different variable importance measure methods (increase in mean square errors and increase in node purity), respectively (Figure 3A).

To clarify the impact of each feature on the outcome prediction, PDPs showed higher values of CRP, dNLR, ALP, WBC, pulse rate, and ECOG and lower values of PD‐L1 expression, ALB, and HGB were each associated with a higher predicted probability of death. Continuous variables (CRP and pulse rate) showed nonlinear associations. In addition, patients with bladder transitional cell carcinoma, liver metastasis, and more than two metastatic sites were associated with a higher predicted probability of death (Figure 3B).

Table 3 provided the univariate model analysis for mortality prediction, the 12 variables individually have varying degrees of contribution to the mortality prediction. A further model was developed by a combination of the 12 LASSO regression selected variables and other variables (e.g., sex, statin use, tumor stage, number of comorbidities, and body mass index). However, the addition of other variables individually to the RF‐integrated 12 features model had no notable improvements in the AUC value on the validation data set (Table 3).

**TABLE 3:** Comparison of each model feature performance with that from the integrated RF model for mortality prediction

Variable	Development cohort AUC (95% CI)	Validation cohort AUC (95% CI)
C‐reactive protein	0.928 (0.917–0.939)	0.602 (0.562–0.642)
Pulse rate	0.632 (0.607–0.658)	0.546 (0.505–0.587)
Cancer type	0.599 (0.577–0.622)	0.604 (0.570–0.638)
PD‐L1 level	0.601 (0.578–0.624)	0.608 (0.573–0.643)
Hemoglobin	0.613 (0.590–0.635)	0.633 (0.599–0.667)
Alkaline phosphatase	0.587 (0.568–0.606)	0.562 (0.534–0.590)
Prior liver metastasis	0.579 (0.560–0.598)	0.576 (0.547–0.604)
Albumin	0.589 (0.568–0.610)	0.589 (0.558–0.621)
White blood cell count	0.569 (0.551–0.587)	0.545 (0.518–0.573)
Number of metastatic sites	0.581 (0.559–0.604)	0.578 (0.544–0.613)
Derived neutrophil/lymphocyte ratio	0.569 (0.552–0.587)	0.559 (0.533–0.586)
ECOG PS	0.578 (0.555–0.600)	0.571 (0.536–0.605)
RF‐integrated 12 features	0.844 (0.826–0.862)	0.786 (0.754–0.818)
RF‐integrated 12 features + Sex	0.849 (0.831–0.866)	0.786 (0.754–0.818)
RF‐integrated 12 features + Statins use	0.843 (0.826–0.861)	0.786 (0.754–0.818)
RF‐integrated 12 features + Tumor stage	0.850 (0.833–0.868)	0.785 (0.752–0.817)
RF‐integrated 12 features + No. of comorbidities	0.849 (0.832–0.867)	0.784 (0.752–0.817)
RF‐integrated 12 features + Body mass index	0.871 (0.855–0.887)	0.782 (0.749–0.814)

Abbreviations: AUC, area under the curve; 95% CI, 95% confidence interval; ECOG PS, Eastern Cooperative Oncology Group performance status; PD‐L1, programmed cell death ligand 1; RF, random forest.

### 3.5 Risk stratification by machine learning associated with prognosis

Herein, the probability with a cutoff at 0.45 was used to assign the predicted mortality risk label by maximizing the F1 score of the random forest algorithm (Figure S2). Probabilities of less than 0.45 were assigned to low risk and otherwise to high risk. Of the 2538 patients in the overall cohort, 1782 (70.2%) were separated into the high‐risk group and 756 (29.8%) into the low‐risk group.

**FIGURE 4:**
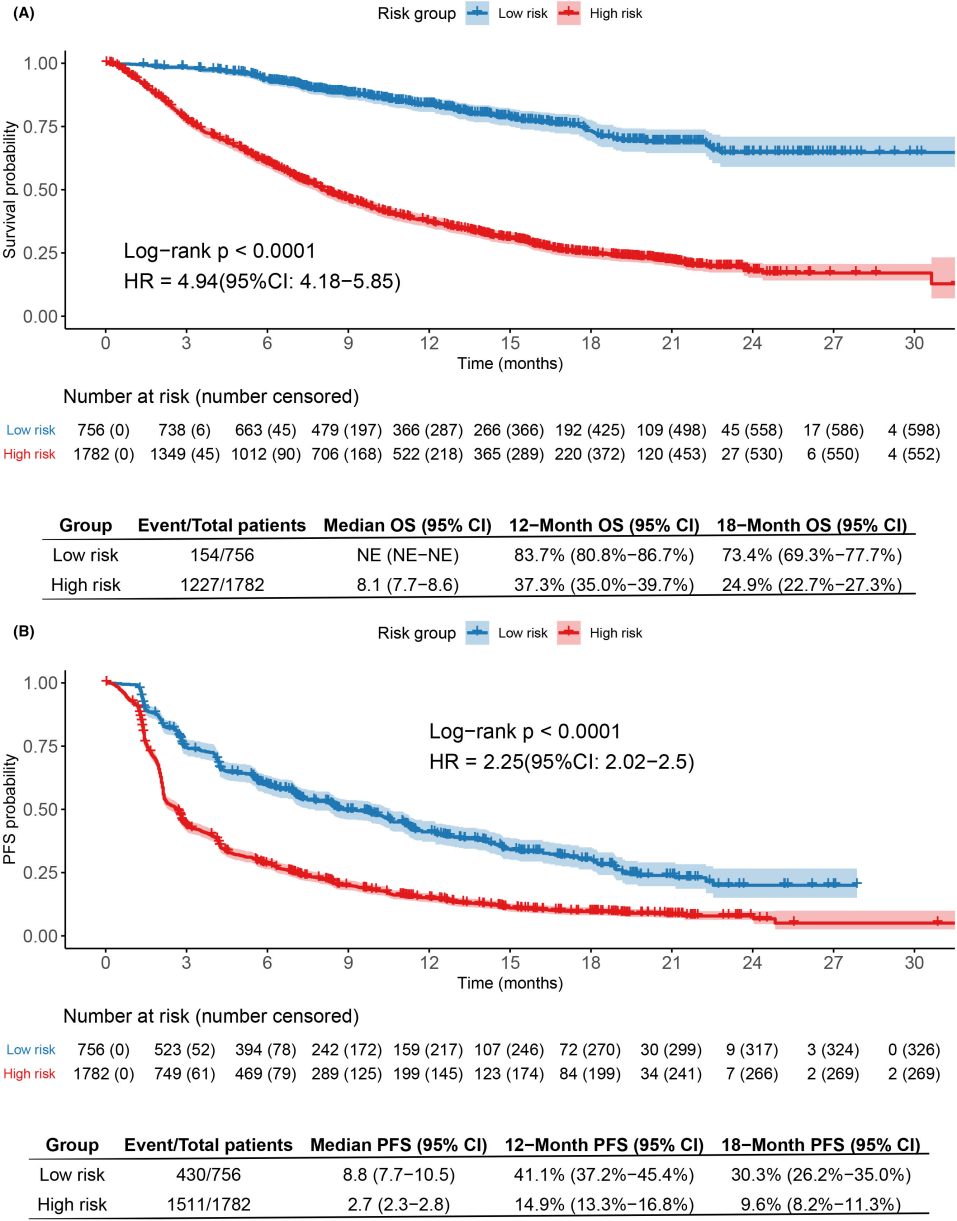
Kaplan–Meier plots indicating differences in survival probability (A) and PFS probability (B) of different risk groups stratified by the random forest model in the whole cohort. 95% CI, 95% confidence interval; HR, hazard ratio; NE, not evaluable; OS, Overall survival; PFS, Progression‐free survival.

As shown in Figure 4A,B, the overall survival and progression‐free survival (PFS) of patients in the whole cohort were significantly stratified by the cutoff value (0.45). High‐risk patients labeled by the RF model were significantly less likely to survive than low‐risk patients in the whole cohort (HR: 4.94; 95% CI: 4.18–5.85; *p* < 0.0001). The median overall survival for patients in the high‐risk group was 8.1 months (95% CI: 7.7–8.6), but the median overall survival was not reached for patients in the low‐risk group. The OS rates in high‐risk group and low‐risk group were 37.3% (95% CI: 35.0%–39.7%) and 83.7% (95% CI: 80.8%–86.7%) at 12 months; 24.9% (95% CI: 22.7%–27.3%), and 73.4% (95%CI: 69.3%–77.7%) at 18 months, respectively (Figure 4A).

The median progression‐free survival (PFS) for the high‐risk group was 2.7 months (95% CI: 2.3–2.8), which was also substantially worse than the median PFS for the low‐risk group (8.8 months [95% CI: 7.7–10.5]; HR: 2.25 [95% CI: 2.02–2.5]; *p* < 0.0001). The 12‐month PFS probability was 14.9% (95% CI: 13.3%–16.8%) for the high‐risk group versus 41.1% (95% CI: 37.2%–45.4%) for the low‐risk group. The 18‐month PFS probability was 9.6% (95% CI: 8.2%–11.3%) for the high‐risk group and 30.3% (95% CI: 26.2%–35.0%) for the low‐risk group (Figure 4B). Of note, the median follow‐up time for survival was 16.2 months (95% CI: 15.7–16.8) in the whole cohort (data not shown).

### 3.6 Secondary outcomes across risk groups

We examined whether there were significant differences in the distribution of secondary outcome events across risk groups. Patients in the high‐risk group were significantly more likely to die, experience disease progression, discontinue study, and discontinue treatment than patients in the low‐risk group (all P values <0.001, Figure S3A–D). The incidence of irAEs, grade 3–5 TRAES was also higher in the high‐risk group versus the low‐risk group, but there was no statistically significant difference (all *p* values > 0.05, Figure S3E,F).

## 4 DISCUSSION

In this study, we built and internally validated an ML‐based risk stratification model to predict mortality of atezolizumab‐treated cancer patients nearly 16.2 months in advance by integrating high‐quality clinical trial data. We found that the random forest model was overall the best in terms of predictive performance.

We identified a set of 12 factors independently associated with mortality among atezolizumab‐treated cancer patients. Although the majority of these factors have been proven correlated with prognosis among cancer patients, including CRP, WBC, neutrophil‐to‐lymphocyte ratio, albumin, alkaline phosphatase, hemoglobin, and ECOG, this is the first study that shows their combined effect when incorporated into one model.^23^, ^24^, ^25^, ^26^ The association of CRP, WBC, and dNLR with mortality has been attributed to their role in inflammation. Prior studies suggested that albumin level may be an important prognostic factor in oncology patients that reflected general nutritional status.^27^ An elevated level of alkaline phosphatase may reflect the liver tumor burden.^28^ The current study confirms that ALP has important prognostic implications in cancer patients. While the potential for PD‐L1 expression to predict the clinical benefit of immunotherapy remains under investigation, this study demonstrates that PD‐L1 expression levels have a significant prognostic role in cancer patients receiving atezolizumab monotherapy.

Cancer type, tumor stage, number of metastatic sites, and ECOG performance status are well‐known factors associated with prognosis in all cancers.^23^, ^29^ Although there was a higher proportion of patients with stage IV cancer who died, tumor stage was not a significant predictor of mortality in our study. Prior liver metastasis was chosen as an important predictor of mortality in our model. A previous study revealed that cancer patients with liver metastasis had reduced response to anti‐PD‐1 immunotherapy and had poor overall survival due to reduced marginal infiltration by CD8 cells.^30^ This evidence supports our result.

Overall, laboratory test variables (CRP, PD‐L1 level, dNLR, ALP, ALB, HGB, and WBC) and tumor characteristics (cancer type, prior liver metastasis, and the number of metastatic sites) are the two major factors for mortality prediction. Thus, laboratory tests and tumor characteristics evaluation performed at baseline before starting immunotherapy may provide an important reference for weighing the benefits and risks of therapy.

This study has several important clinical implications. To our knowledge, this study is the first to exclusively apply ML algorithms for mortality prediction in cancer patients initiating immunotherapy. It is suggested that high‐risk patients classified by our RF model, accounting for about 70.2% of the study population, should be considered for more advanced treatment due to poor prognosis. Beyond mortality prediction, our study has extended the application of the ML‐based risk stratification model for predicting disease progression, discontinued study, and discontinued treatment. Additionally, the ML‐based risk stratification model has the potential to help identify benefiting patients in clinical trials.^31^ Our RF model successfully identifies a subset of patients (29.8%) that benefits from atezolizumab monotherapy treatment.

Furthermore, our analysis provides insights into the relative contributions of 12 predictors of death. None of the 12 predictors alone could reach the level of performance obtained by the RF‐integrated 12 features model, indicating that a nonlinear combination of multiple factors contributed to various degrees to the overall prediction performance. This finding is concordant with a previous report from Chowell and colleagues.^32^ Our modeling principle is a trade‐off between having a minimal number of variables and good prediction performance. The prediction accuracy of an algorithm depends heavily on the presence of relevant predictors. Of note, ML‐based models performed better than conventional logistic regression‐based models. To avoid overfitting and improve the stability of the model, internal validations were conducted via a fivefold cross‐validation approach for the best‐performing model in this study. The AUC of the random forest algorithm across five validation experiments ranged from 0.757 to 0.786. These results imply little variation and that the model does not overfit the data—a characteristic of good machine‐learning models. The 12 variables for outcome prediction were readily available and frequently tested in routine clinical practice, which might increase the feasibility of implementing the predictive model in clinical practice.

Our study has several limitations. First, this was a post‐hoc pooled analysis of clinical trials data, and there may be potential selection bias. We did not analyze the patient‐reported outcome (PRO) data as this information was not available in some trials (ClinicalTrials.gov Identifier: NCT02951767; NCT02108652), although baseline PRO variables have already been described as a predictor of survival.^33^ Additionally, we lacked biomarker data such as tumor mutational burden (TMB) that is potentially related to patient response to immunotherapy.^8^, ^32^ If available, they may improve predictive performance even further. Second, the primary limitation of ML algorithms is that they are viewed as a “black box,” and it might be hard to interpret all kinds of nonlinear relationships among variables. Although this study identified the most important variables with respect to predicting mortality, these feature relationships should be interpreted with caution. Third, our model was developed on data from patients treated with atezolizumab monotherapy and is thus unlikely to be accurate for patients receiving other ICIs or ICI combination therapy. The prognostic profiles in cancer patients may vary depending on the type of immune checkpoint inhibitors. Of note, for each specific immunotherapy agent, predictive models may have different variables that predict mortality. Fourth, our study population involved three cancer types (non‐small‐cell lung cancer, bladder transitional cell carcinoma, and renal cell carcinoma), so it is possible that the results may not be completely generalizable to other cancer types. Further studies are required to externally validate the model and should explore how to best optimize mortality prediction models for patients receiving different immunotherapy agents across multiple cancer types.

In summary, this study demonstrates the clinical implication and value of the ML‐based model for risk stratification of mortality as early as the time of treatment initiation. We believe that this model with 12 variables could be a useful tool to identify individuals who are most likely to have adverse outcomes (death, disease progression, discontinued study, or discontinued treatment) and clinical benefit from atezolizumab monotherapy.

## AUTHORS' CONTRIBUTION

Conceptualization: Y.G.W., W.Z., J.D.S., and Y.W.; Data curation: W.Y.Z., J.W., and Y.G.W.; Formal analysis: W.Y.Z., J.W., and Y.G.W.; Investigation: J.D.S. and Y.W.; Methodology: W.Y.Z., J.W., and Y.G.W.; Project administration: Q.Q.Q., Y.H., J.X., and Y.T.G.; Software: W.Y.Z., J.W., and Y.G.W.; Supervision: Q.Q.Q., L.W.L., and Y.H.; Validation: Y.G.W and W.Y.Z.; Visualization: J.X. and Y.T.G.; Writing—original draft: Y.G.W.; Writing—review and editing: Y.G.W., W.Y.Z., J.W., and L.W.L. All the authors have read and agreed to the published version of the manuscript.

## CONFLICT OF INTEREST

The authors declare that they have no competing interests.

## ETHICS STATEMENT

Ethical approval was waived by the local institutional review board or ethics committee, as secondary analysis of publicly available anonymized patient‐level trial data was deemed to be a negligible risk.

## Supporting information


Appendix S1

## Data Availability

Data were accessed according to Roche's policy and process for clinical study data sharing and are available for request at https://vivli.org/.
