# Supplementary material for: Using machine learning for mortality prediction and risk stratification in atezolizumab‐treated cancer patients: Integrative analysis of eight clinical trials
Source: Cancer Med. 2022 Jul 24;12(3):3744–57. doi: 10.1002/cam4.5060 (PMC9939114; doi:10.1002/cam4.5060)
Supplement: Supplementary file 1 — Appendix S1 [file CAM4-12-3744-s001.docx]

| Supplemental Table 1: Descriptive information about clinical trials used in this study | | | | | | | | |
| --- | --- | --- | --- | --- | --- | --- | --- | --- |
| Study | ROCHE-GO28625 | ROCHE-GO28753 | ROCHE-GO28754 | ROCHE-GO28915 | ROCHE-GO29293 (cohort 1) | ROCHE-GO29293 (cohort 2) | ROCHE-GO29294 | ROCHE-WO29074 |
| ClinicalTrials.gov identifier | NCT01846416 | NCT01903993 | NCT02031458 | NCT02008227 | NCT02951767 | NCT02108652 | NCT02302807 | NCT01984242 |
| Cancer type | NSCLC | NSCLC | NSCLC | NSCLC | BTCC | BTCC | BTCC | RCC |
| Study phase | 2 | 2 | 2 | 3 | 2 | 2 | 3 | 2 |
| Stage | IIIB, IV | IIIB, IV | IIIB, IV | IIIB, IV | IV | IV | IV | IV |
| Experimental arm/ Number of patients | Atezolizumab/138 | Atezolizumab/ 144 | Atezolizumab/667 | Atezolizumab/ 613 | Atezolizumab/119 | Atezolizumab/ 310 | Atezolizumab/ 467 | Atezolizumab/ 103, Atezolizumab and Bevacizumab/ 101 |
| Comparator arm/ Number of patients | NA/NA | Docetaxel/ 143 | NA/NA | Docetaxel/ 612 | NA/NA | NA/NA | Chemotherapy/464 | Sunitinib/ 101 |
| Inclusion criteria in brief | Stage IIIB, Stage IV, or recurrent NSCLC, PD-L1-positive status, ECOG performance status of 0 or 1 | Stage IIIB, Stage IV, or recurrent NSCLC with disease progression during or following treatment with a prior platinum-containing regimen, ECOG performance status of 0 or 1 | Stage IIIB, Stage IV, or recurrent NSCLC, PD-L1-positive status, ECOG performance status of 0 or 1 | Stage IIIB, Stage IV, or recurrent NSCLC with disease progression during or following treatment with a prior platinum-containing regimen, ECOG performance status of 0 or 1 | Locally advanced or metastatic or recurrent urothelial carcinoma with no prior chemotherapy, ECOG performance status of 0 or 1 | Locally advanced or metastatic urothelial bladder cancer with disease progression following treatment with at least one platinum-containing regimen, ECOG performance status of 0 or 1 | Locally advanced or metastatic urothelial bladder cancer with disease progression following treatment with at least one platinum-containing regimen, ECOG performance status of 0 or 1 | Unresectable advanced or metastatic renal cell carcinoma with no prior systemic therapy |
| Primary outcome | Objective response rate | Overall survival | Objective response rate | Overall survival | Objective response rate | Objective response rate | Overall Survival | Progression-Free Survival |
| Secondary outcome | Progression-Free Survival, Overall Survival | Objective Response Rate, Progression-Free Survival | Progression-Free Survival, Overall Survival | Progression-Free Survival | Progression-Free Survival, Overall Survival | Progression-Free Survival, Overall Survival | Progression-Free Survival, Objective response rate | Objective response rate, Overall Survival |
| Line of therapy | First or second line | NA | First, second or third line | NA | First line | Second-line or beyond treatments | NA | NA |
| Abbreviations: NSCLC, non-small cell lung cancer; BTCC, bladder transitional cell carcinoma; RCC, renal cell carcinoma; NA, not applicable. | | | | | | | | |
|  |  |  |  |  |  |  |  |  |


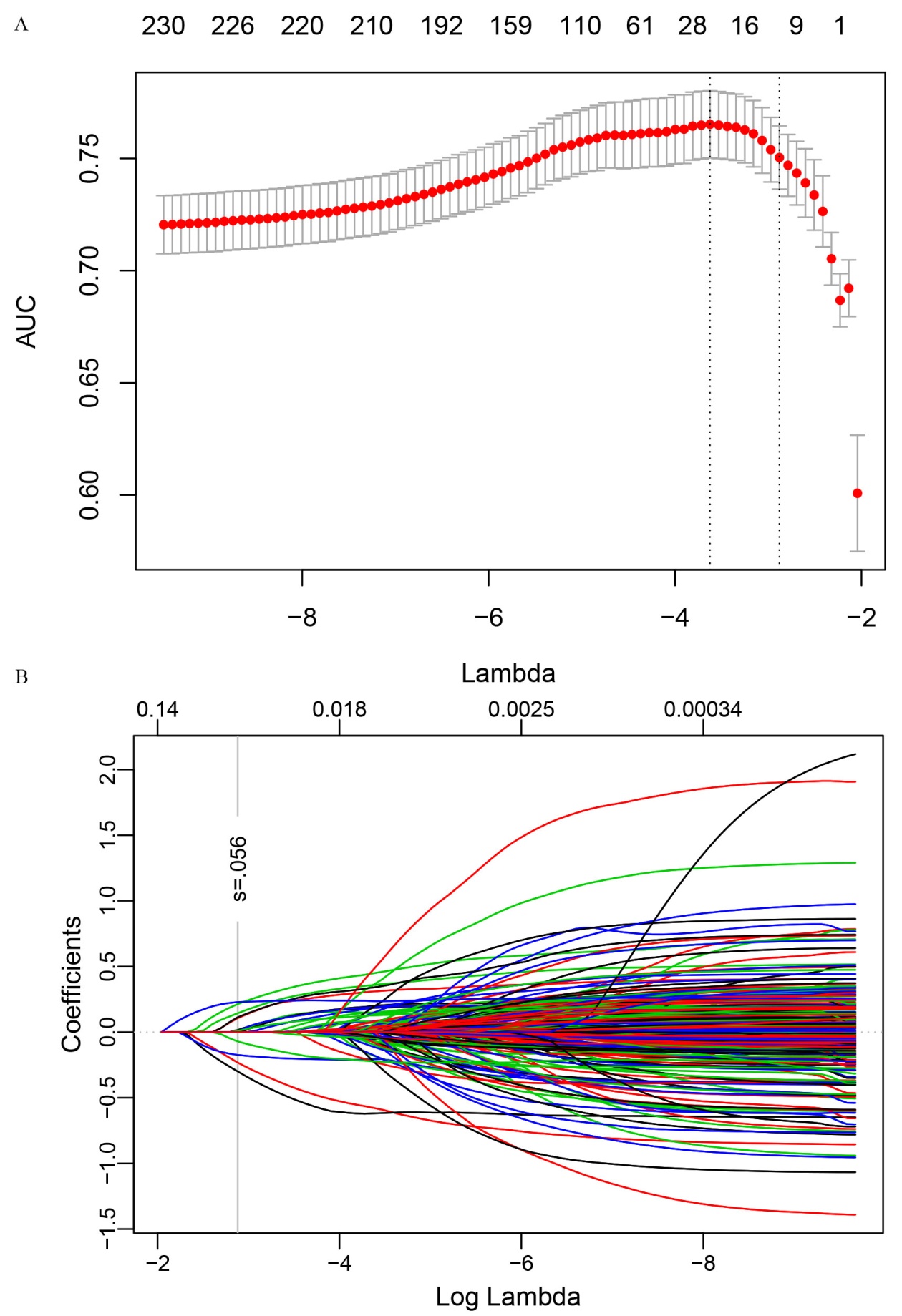


**Supplementary Figure 1.** Feature selection using the least absolute shrinkage and selection operator (LASSO) binary logistic regression model. (A) Tuning parameter lambda (λ) selection in the LASSO model using 10-fold cross-validation via minimum criteria. The area under the receiver operating characteristic (AUC) curve was plotted versus log (lambda). Two vertical dashed lines were drawn at the optimal values by using the minimum criteria (lambda.min) and the 1 standard error of the minimum criteria (lambda.1se). (B) LASSO coefficient profile plot was produced against the lambda sequence. The vertical line shows the optimal lambda value 0.056 (lambda.1se) chosen by tenfold cross validation, where optimal lambda yielded 12 predictors with nonzero coefficients.


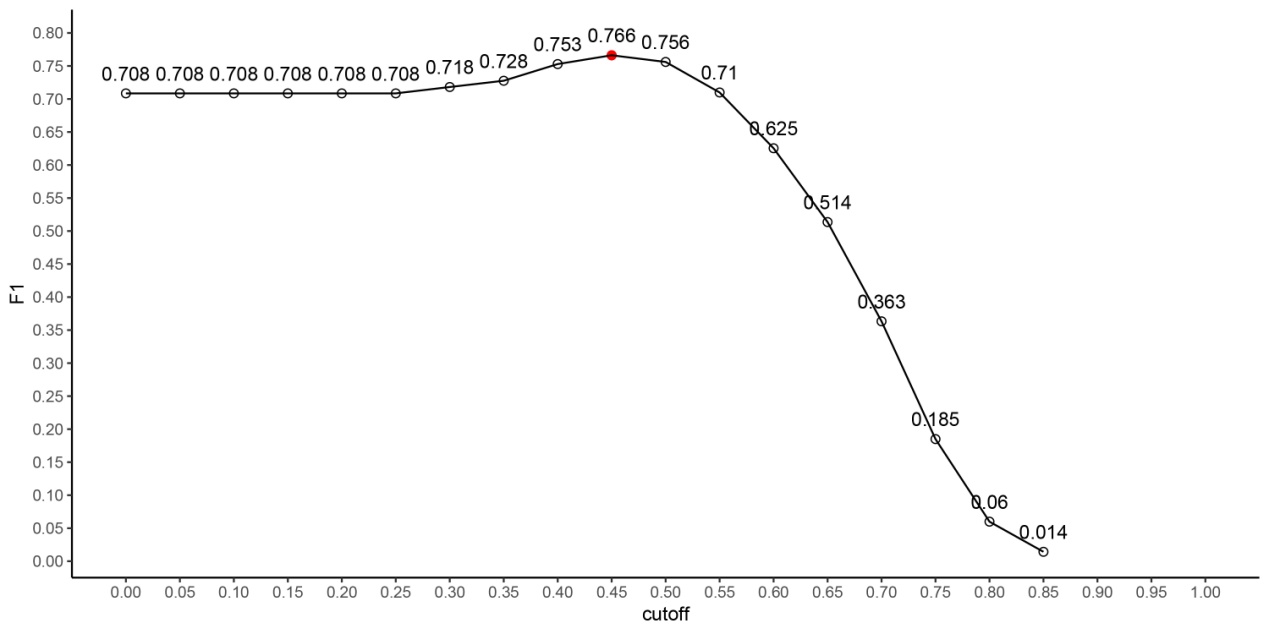


**Supplementary Figure 2.** F1 score of random forest model on validation cohort in respect to different probability cutoff value.


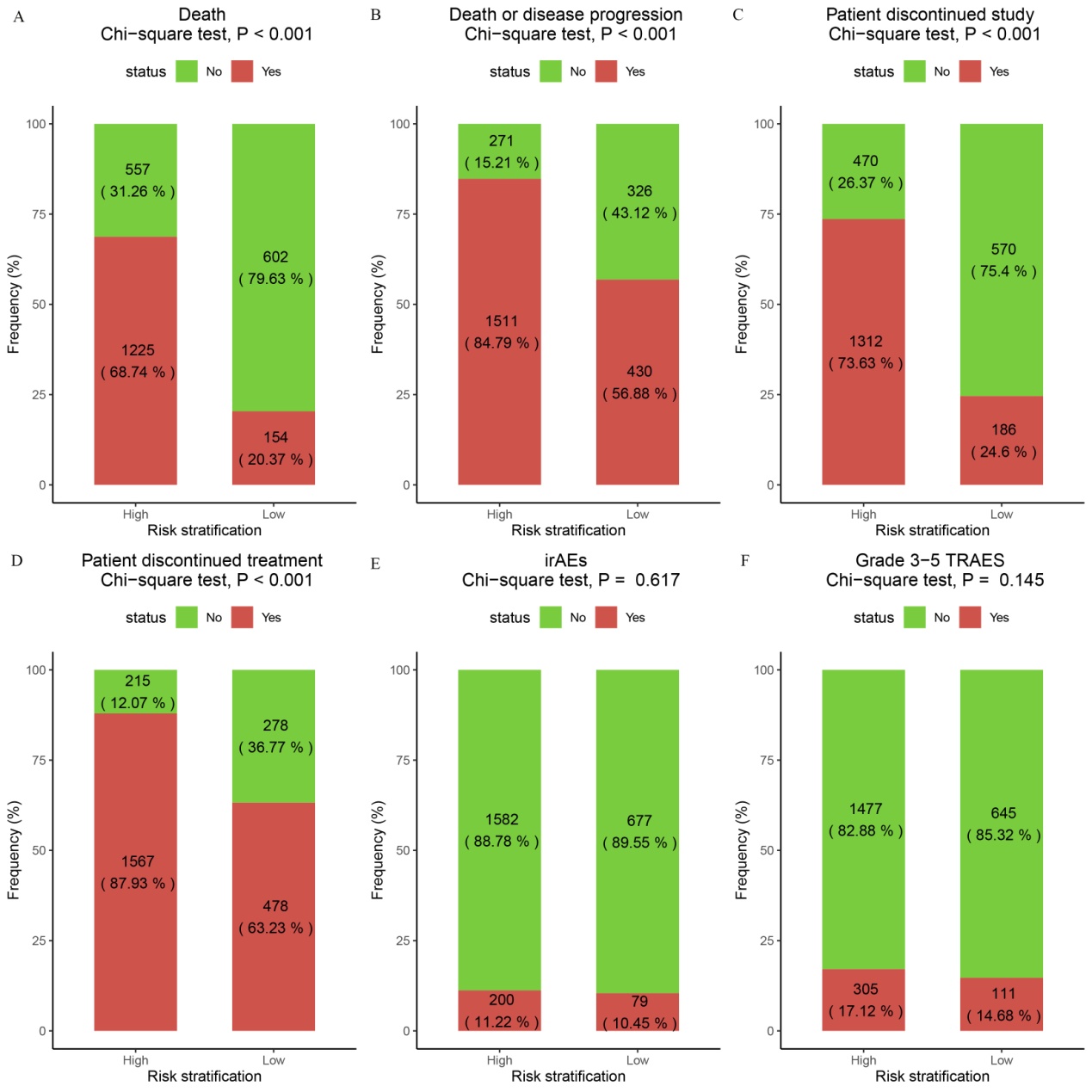


**Supplementary Figure 3.** Association of risk stratification with the proportion of patients observed to undergo death (A), death or disease progression (B), discontinued study (C), discontinued treatment (D), irAEs (E), grade 3-5 TRAES (F) as observed in the overall cohort. irAEs, Immune-related adverse events; TRAES, Treatment-related adverse events.
